# Casper Versus Precise Stent for the Treatment of Patients with Idiopathic Intracranial Hypertension

**DOI:** 10.1007/s00062-021-01024-2

**Published:** 2021-05-18

**Authors:** Nebiyat F. Belachew, Severin Baschung, William Almiri, Ruben Encinas, Johannes Kaesmacher, Tomas Dobrocky, Christoph J. Schankin, Mathias Abegg, Eike I. Piechowiak, Andreas Raabe, Jan Gralla, Pasquale Mordasini

**Affiliations:** 1 Department of Diagnostic and Interventional Neuroradiology, Inselspital, Bern University Hospital, and University of Bern, Freiburgstraße 18, 3010 Bern, Switzerland; 2 Faculty of Medicine, University of Bern, Bern, Switzerland; 3 Department of Diagnostic, Interventional and Pediatric Radiology, Inselspital, Bern University Hospital, and University of Bern, Bern, Switzerland; 4 Department of Neurology, Inselspital, Bern University Hospital, and University of Bern, Bern, Switzerland; 5 Department of Ophthalmology, Inselspital, Bern University Hospital, and University of Bern, Bern, Switzerland; 6 Department of Neurosurgery, Inselspital, Bern University Hospital, and University of Bern, Bern, Switzerland

**Keywords:** Pseudotumor cerebri, Venous sinus stenting, Venous sinus stenosis, Cerebrospinal fluid opening pressure, Stent delivery

## Abstract

**Purpose:**

We hypothesized that due to its specific characteristics, the Casper^TM^ RX carotid stent (CP) might be particularly suitable for venous sinus stenting (VSS) in patients with idiopathic intracranial hypertension (IIH). To test this theory, we compared it to the commonly used Precise Pro RX^TM^ stent (PP).

**Methods:**

A total of 15 patients with IIH (median age 28.7 years) were reviewed retrospectively. Technical aspects as well as peri- and postinterventional complication rates were examined in patients treated with CP (*n* = 10) and the PP (*n* = 5). Improvements in cerebrospinal fluid opening pressure (CSF OP), transstenotic pressure gradient (TSPG) and clinical symptoms were also assessed.

**Results:**

Stent delivery was easier and more successful with the CP than the PP (difficult/failed stent delivery 0.0% versus 57.1%) and consequently achieved with less attempts (≥ 2: 0.0% versus 40.0%). No severe peri- or postinterventional complications or instances of in-stent thrombosis and/or stenosis were observed during follow-up. Improvement of CSF OP and TSPG immediately after VSS as well as at 6‑month follow-up was comparable between the CP and PP group. Both groups showed substantial and similar decreases in intensity and frequency of headache. Almost all patients with other IIH-related symptoms showed either improvement or complete resolution of those symptoms after VSS. All patients who were available for interview (*n* = 12/15) reported a substantial improvement in quality of life.

**Conclusion:**

VSS using the CP seems to be safe and effective. The CP may reduce the risk of difficult or failed stent delivery in patients with challenging intracranial venous anatomy.

**Supplementary Information:**

The online version of this article (10.1007/s00062-021-01024-2) contains supplementary material, which is available to authorized users.

## Introduction

Idiopathic intracranial hypertension (IIH) is a relatively rare disease mainly affecting obese women of childbearing age (12–20 per 100,000 people per year in this group [1]). Patients with IIH often exhibit a series of chronic symptoms, such as moderate to severe headache, nausea/vomiting, pulse synchronous tinnitus, photophobia/phonophobia, diplopia and other visual disturbances (e.g. obscuration, visual field defects) [2]. The increased intracranial pressure observed in patients with IIH may lead to permanent damage of the optic nerve and fulminant vision loss [2]. Venous sinus stenting (VSS) has emerged as a promising and efficient treatment alternative for IIH patients whose condition is not improved by conservative treatment and who demonstrate a functionally relevant venous sinus stenosis [3–9]; however, device delivery may be challenging owing to the tortuosity of the transverse and sigmoid sinuses, the high degree of stenosis, the angle of the stenotic segment, small venous channels within the sinus, arachnoid granulations, fibrous trabeculae or the presence of a cortical vein draining into the dural venous sinuses, which are frequently observed in IIH patients [10–13]. Forced device maneuvers can lead to severe hemorrhagic complications including avulsion of cortical veins and dissection or perforation of the sinus [14–16]. Since dedicated stent systems for this indication are not available VSS is often performed by off-label application of carotid stents [17]. The Casper RX^TM^ (CP) stent system (Casper RX^TM^, Microvention, Terumo, Tustin, CA, USA) is a self-expanding, braided, nitinol stent with a dual-layer micromesh design approved for carotid artery stenting. The stent design is aimed to improve coverage, flexibility, conformability and wall apposition of the device, which should lower the risk of periinterventional stroke during carotid artery stenting procedures. We aimed to examine whether these particular stent characteristics provide a technical advantage compared to another commonly used stent devices [18].

## Material and Methods

### Inclusion Criteria

The clinical and neurointerventional data presented in this study were collected by reviewing medical records as well as interviewing IIH patients who underwent VSS at Bern University Hospital between October 2016 and April 2020. The inclusion criteria were as follows: (1) final diagnosis of IIH according to the modified Dandy criteria [19], (2) confirmation of a functionally relevant venous sinus stenosis and (3) VSS performed. A venous sinus stenosis was considered functionally relevant if the transstenotic pressure gradient (TSPG) was ≥ 4 mm Hg. Patients were regarded as eligible for VSS if they were deemed refractory to conservative treatment by the treating physicians or treatment had to be stopped owing to side effects. Emergency VSS was performed in cases of sudden and severe vision impairment in conjunction with a functionally relevant venous sinus stenosis confirmed with venous sinus manometry. General consent was obtained from all 15 patients. This study was approved by the local ethics committee.

### Analysis of Clinical Information

Baseline demographics and clinical information, such as age, sex, arterial hypertension, diabetes mellitus and obesity (including body mass index) were gathered. The IIH-related symptoms that were present prior to VSS, such as headache [20] (intensity according to the visual analogue scale, VAS 0–10 and frequency per week), tinnitus of any kind, phonophobia and photophobia, nausea and/or emesis, diplopia and other subjective visual disturbances (e.g. transient visual obscuration, blurred vision) were also documented. Patients who experienced headaches of more than one type in terms of severity, development, localization and expansion were evaluated according to the instructions provided in the supplementary form A. The time from symptom onset to first treatment as well as the duration of conservative treatment were documented. Finally, the presence of papilledema and the cerebrospinal fluid opening pressure (CSF OP) prior to VSS was recorded.

### DSA and Venous Sinus Manometry

Venous sinus stenosis was diagnosed on digital subtraction angiography (DSA) and then quantified with venous sinus manometry (VSM) with the patient under local anesthesia (LA). After establishing a transfemoral arterial and venous access, a 6F guiding catheter was positioned in the jugular bulb while the diagnostic catheter was placed in the ipsilateral carotid artery. A VSM was then performed by navigating a 0.027inch microcatheter through the venous sinus and measuring the TSPG as described by Fargen et al. [21].

### Venous Sinus Stenting

Patients eligible for VSS were administered 100 mg/day aspirin and 75 mg/day clopidogrel starting at least 5 days prior to treatment. The DSA and VSM were repeated immediately prior to VSS with the patient under general anesthesia (GA). The VSS was performed according to institutional protocols and in accordance with the recommendation published by Fargen et al. [22]. We used a standardized endovascular access approach in all interventions. Venous access was gained through the right common femoral vein by inserting an 8F 25 cm sheath (Radifocus® Introducer II, Terumo, Tokyo, Japan). Then, an 8F guiding catheter (Guider Softip^TM^, Boston Scientific, Marlborough, USA; Neuron MAX^TM^ 088, Penumbra, Almeda, CA, USA) was advanced as distally as possible into the ipsilateral internal jugular vein or even the sigmoid sinus if possible. If stent delivery was not possible by this monoaxial approach (e.g. because of increased resistance and loading of the system), the stent was delivered through an additional intermediate catheter (Vasco +35, BALT, Montmorency, France; 6F Sofia, Microvention, Tustin, USA), which was advanced as distally as possible in order to achieve better pushability and deliverability. A long-braided sheath (8F Super Arrow-Flex PSI set 80 cm, Arrow International, Reading, PA, USA) was introduced into the internal jugular vein to further increase proximal stability, if necessary. The decision on which stent to use was not randomized and was made by the attending neurointerventionalist based on the patient’s intracranial venous anatomy and the physician’s personal experience. The difficulty of stent delivery was assessed retrospectively by a neurointerventionalist (P.M.) with 12 years of experience after reviewing the DSA images and the corresponding neurointerventional report. Stent delivery was classified as easy in cases of monoaxial access suggesting minimal loading of the system and/or no mention of difficulties in the interventional report, as difficult in the case of a biaxial access using an intermediate catheter and/or a long-braided sheath suggesting increased loading of the system and/or mention of difficulties in the interventional report or as failed if stent delivery could not be performed using the initial endovascular approach. The number of maneuvers required for successful stent delivery were documented. Balloon-assisted dilatation after stent deployment was performed in cases of residual stenosis, which was considered relevant if the TSPG remained ≥ 4 mm Hg. At the end of the procedure, DSA and VSM were repeated to screen for periprocedural complications and to compare preinterventional and postinterventional transstenotic pressure values. All patients were monitored in an intermediate care unit for a minimum of 24 h after VSS before being transferred to the neurology ward. Aspirin, 100 mg/day, was continued lifelong and clopidogrel, 75 mg/day, for 6 months after VSS.

### Stent Systems

All patients were treated with either the CP or the Precise Pro RX^TM^ stent (PP). The PP (CardinalHealth, Cordis, Santa Clara, CA, USA) is a self-expanding, laser-cut nitinol stent with a single-layer, V‑pattern open-cell design, developed for the treatment of carotid artery stenosis. According to the literature, it is one of the stent devices most commonly used off-label for VSS in patients with IIH [17]. Compared to the CP, the open-cell V‑segments in the PP have a larger strut size. Although the single-layer, open-cell design makes the delivery system more rigid than the CP, it provides a smaller contact surface, which may reduce thrombogenicity and the risk of venous occlusion.

The CP (Microvention, Terumo, Tustin, CA, USA) is a self-expanding, braided, nitinol stent with a dual-layer micromesh design, originally developed to treat patients with carotid artery stenosis [23]. Its inner layer has small cells measuring 375–500 µm, which are meant to reduce the risk of plaque prolapse and embolic release [24]. The nitinol material, the braided structure and the low profile provide a high level of flexibility and crossability allowing the stent to adapt to tortuous vessel anatomy without being prone to kinking [24]. The CP can be resheathed and repositioned after up to 50% deployment, which enables a more accurate device placement.

### Follow-up at 6 Months and Outcome

The DSA, VSM and diagnostic lumbar puncture for CSF OP measurement were repeated under LA at the 6‑month follow-up. In addition to chart review, patients were interviewed at least 6 months after VSS to assess improvement of all IIH-related symptoms as well as quality of daily life (see Supplementary form A). Patients were also asked about their symptoms prior to VSS. Their statements were compared to the information acquired from chart review. The interval between VSS and the last clinical follow-up was documented for each patient. Evolution and development of papilledema was assessed by reviewing ophthalmology reports. Postinterventional magnetic resonance imaging (MRI) and clinical charts were checked for any signs of intracranial hemorrhage, venous infarction or other signs of postinterventional complications.

### Statistical Analysis

Data analysis was performed using SPSS Software (Version 25.0, IBM Analytics, Armonk, NY, USA). Continuous variables were compared using the Mann-Whitney U‑test, whereas categorical variables were compared with Fischer’s exact test. Results with two-tailed *p*-values of < 0.05 were considered statistically significant and are shown as median (interquartile range 25–75%), median comparisons with *p*-values according to applied test or total values (*n*).

## Results

Between October 2016 and April 2020 there were 15 female patients (mean ag: 28.7 years, range 24.0–41.5 years) diagnosed with IIH who underwent VSS for venous sinus stenosis at Bern University Hospital. There were no differences in demographic characteristics or comorbidities between patients treated with the CP (*n* = 10) and the PP (*n* = 5) (Table 1). One patient who was scheduled to receive the PP was ultimately treated with the CP due to failure of delivery of the PP. For the purpose of comparing postinterventional outcome parameters, this patient was included in the CP group. Prior to VSS, 14 patients had been managed conservatively and 1 patient (*n* = 1/15; in the PP group) underwent surgery (ventriculoperitoneal shunt). Mean duration of conservative treatment tended to be longer but not statistically significantly different for patients treated with the CP than for patients who received the PP (18.5 months versus 7 months; *p* = 0.679). All venous stenoses treated were located in the lateral segment of the transverse sinus or the transverse-sigmoid junction. Symptoms and pressure values before and after VSS are summarized in Tables 2 and 3.

**Table 1:** Patient demographic characteristics and comorbidities. Data are expressed as percentages (*n*) or median (interquartile range 25–75%)

	Data available for % (*n*)	All patients (*n* = 15)	Casper^TM^ RX stent (*n* = 10)	Precise Pro RX^TM^ stent (*n* = 5)	*P*-value
Age (years)	100% (15/15)	28.7 (24.0–41.5)	27.5 (22.33–37.23)	34.7 (25.45–50.8)	0.310
Sex (female, %)	100% (15/15)	100% (15)	100% (10)	100% (5)	*
**Comorbidities**					
Diabetes mellitus	100% (15/15)	20.0% (3)	10.0% (1)	40.0% (2)	0.242
Arterial hypertension	100% (15/15)	26.7% (4/15)	20.0% (2)	40.0% (2)	0.560
Body mass index	100% (15/15)	30.61 (25.71–34.96)	29.31 (25.63–34.78)	31.71 (26.01–41.90)	0.440
*Obesity*	*100% (15/15)*	*–*	*–*	*–*	*1.000*
None	–	13.3% (2/15)	10.0% (1)	20.0% (1)	–
Moderate	–	33.3% (5/15)	40.0% (4)	20.0% (1)	–
Severe	–	53.3% (8/15)	50.0% (5)	60.0% (3)	–

* No *p*-value as only one variable is present

**Table 2:** Treatment, symptoms and pressure values before VSS. Data are expressed as percentages (*n*) or median (interquartile range 25–75%)

	Data available for % (*n*)	All patients (*n* = 15)	Casper^TM^ RX stent (*n* = 10)	Precise Pro RX^TM^ stent (*n* = 5)	*P*-Value
**Headache**					
Headache intensity (VAS)	80.0% (12/15)	7.25 (5.88–9.0)	8.75 (7.13–9.38)	6.25 (1.38–7.0)	0.048^a^
*Headache frequency (per week)*	*80.0% (12/15)*	*7 (5.13–7)*	*7 (5.13–7)*	*7 (1.8–7)*	*0.933*
≤ 1	–	13.4% (2)	10.0% (1)	20.0% (1)	–
2–4	–	0.0% (0)	0.0% (0)	0.0% (0)	–
> 4	–	66.7% (10)	70.0% (7)	60.0% (3)	–
Presence of more than one type of headache	80.0% (12/15)	46.7% (7)	40.0% (4)	60.0% (3)	0.576
**Other symptoms/findings**					
Nausea/vomiting	100% (15/15)	66.7 (10/15)	80.0% (8)	40.0% (2)	0.251
Photophobia/phonophobia	100% (15/15)	26.7% (4/15)	30.0% (3)	20.0% (1)	1.000
Tinnitus	100% (15/15)	60.0% (9/15)	50.0% (5)	80.0% (4)	0.580
Diplopia	100% (15/15)	20.0% (3/15)	20.0% (2)	20.0% (1)	1.000
Visual disturbances	100% (15/15)	73.3% (11/15)	60.0% (6)	100% (5)	0.231
Papilledema	100% (15/15)	66.7% (10/15)	60.0% (6)	80.0% (4)	0.600
Daily life impairment	80.0% (12/15)	80.0% (12/15)	80.0% (8/10)	80.0% (4/5)	*
Symptom duration (months)	100% (15/15)	23.6 (4.73–61.67)	26.9 (3.92–102.85)	12 (5.47–170.09)	0.953
**Treatment before VSS**					
Conservative treatment	100% (15/15)	93.3% (14/15)	90.0% (9)	100% (5)	1.000
Duration of conservative treatment (months)	100% (15/15)	18 (6–23)	18.5 (6.0–24.5)	7 (6–27)	0.679
Surgical treatment	100% (15/15)	6.7% (1/15)	0.0% (0)	20.0% (1)	0.333
**Pressure values**					
CSF OP (cmH_2_0)	86.7% (13/15)	31 (24–43.5)	35 (26.0–49.5)	27 (23.0–36.25)	0.414
TSPG on diagnosis in LA (mm Hg)	93.3% (14/15)	22.5 (11.25–26.25)	23 (8.5–26.25)	21.5 (17.75–31.25)	0.733
TSPG immediately before stenting in GA (mm Hg)	100% (15/15)	10 (7–19)	8.5 (2.5–12)	19 (8.5–39)	0.099

*CSF OP* cerebral spinal fluid opening pressure, *GA* general anesthesia, *LA* local anesthesia, *TSPG* transstenotic pressure gradient, *VAS* visual analogue scale, *VSS* venous sinus stenting

* No *p*-value as only one variable is present

**Table 3:** Symptoms and pressure values after VSS. Data are expressed as percentages (*n*) or median (interquartile range 25–75%)

	Data available for % (*n*)	All patients (*n* = 15)	Casper^TM^ RX stent (*n* = 10)	Precise Pro RX^TM^ stent (*n* = 5)	*p*-value
**Headache**					
Headache intensity (VAS)	80.0% (12/15)	0 (0–0)	0 (0–0)	0 (0–1.13)	0.808
Headache intensity improvement (VAS)	80.0% (12/15)	−7 (−8.88 to −4.13)	−8 (−9.38 to −5.13)	−5.5 (−7 to −1)	0.109
*Headache frequency (per week)*	*80.0% (12/15)*	*0 (0–0)*	*0 (0–0)*	*0 (0–0)*	*0.808*
≤ 1	–	73.3% (11)	70.0% (7)	80.0% (4)	–
2–4	–	6.7% (1)	10.0% (1)	0.0% (0)	–
> 4	–	0.0% (0)	0.0% (0)	0.0% (0)	–
Headache frequency improvement (per week)	80.0% (12/15)	−7 (−7 to −4.63)	−7 (−7 to −4.63)	−7 (−7 to −1.78)	0.933
**Improvement of other symptoms/findings among affected**					
Nausea/vomiting	100% (10/10)	100% (10/10)	100% (8/8)	100.0% (2/2)	*
Photophobia/phonophobia	100% (4/4)	100% (4/4)	100% (3/3)	100.0% (1/1)	*
Tinnitus	100% (9/9)	77.8% (7/9)	100% (5/5)	50.0% (2/4)	0.073
Diplopia	100% (3/3)	100% (3/3)	100% (2/2)	100.0% (1/1)	*
Visual disturbances	100% (11/11)	90.9% (10/11)	100% (6/6)	80.0% (4/5)	0.251
Papilledema	90.0% (9/10)	90.0% (9/10)	83.3% (5/6)	100.0% (4/4)	0.153
Substantial improvement of daily life	80.0% (12/15)	100% (12/12)	100% (8/8)	100.0% (4/4)	*
Follow-up period (months)	80.0% (12/15)	16.6 (7.0–31.4)	11.5 (5.4–28.4)	30.47 (11.79–39.22)	0.206
**Pressure values and pressure value improvement**					
CSF OP (cmH_2_0)	80.0% (12/15)	21.0 (16.25–27.5)	22.0 (16.5–29.5)	20 (15–**)	0.373
CSF OP improvement (cmH_2_0)	80.0% (12/15)	−11 (−24 to −6.5)	−12 (−27.5 to −5.5)	−10 (−18–**)	1.000
TSPG immediately after stenting (mm Hg)	100% (15/15)	1 (0–3)	1 (0.75–2.25)	1 (0–5.5)	0.953
TSPG improvement immediately after stenting (mm Hg)	100% (15/15)	−8 (−19 to −5)	−8 (−10.25 to −1.75)	−19 (−35.5 to −6)	0.165
TSPG at 6 months follow-up (mm Hg)	93.3% (14/15)	1 (0–5)	1.5 (0.75–5)	0.5 (0–5.5)	0.454
TSPG improvement at 6 months follow-up (mm Hg)	93.3% (14/15)	−19 (−22.25 to−11)	−19 (−21.25 to −7.75)	−21 (−26 to −17.5)	0.304

*CSF OP* cerebral spinal fluid opening pressure, *GA* general anesthesia, *LA* local anesthesia, *TSPG* transstenotic pressure gradient, *VAS* visual analogue scale, *VSS* venous sinus stenting

* No *p*-value as only one variable is present, ** no 75% percentile

### Difficulty of Stent Delivery and Technical Outcome

**Fig. 1:**
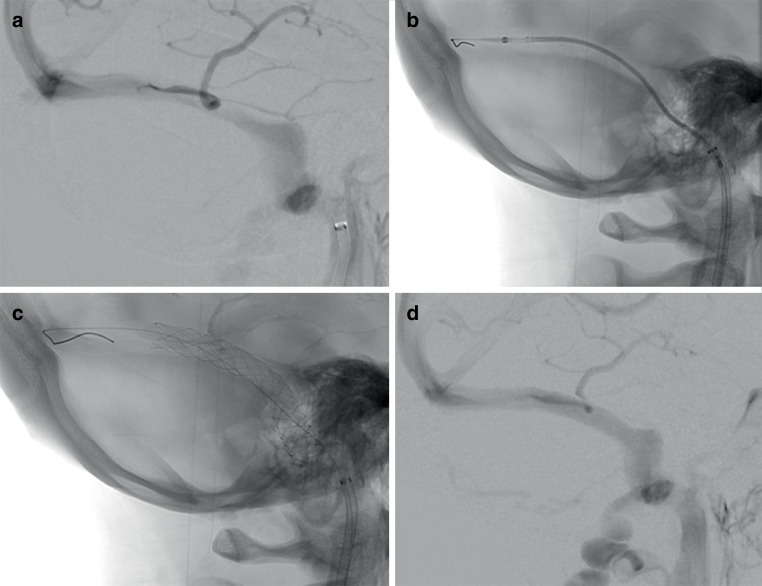
**a** Shows the digital subtraction angiography (DSA) of a patient who has idiopathic intracranial hypertension with a long extrinsic venous sinus stenosis involving the right transverse sinus and the proximal sigmoid sinus. The images that follow show the advancing (**b**) and subsequent deployment (**c**) of a Casper^TM^ carotid stent (10 × 30 mm) across the stenosis without difficulty. The control angiography after stent deployment (**d**) shows no residual stenosis and good wall apposition

**Fig. 2:**
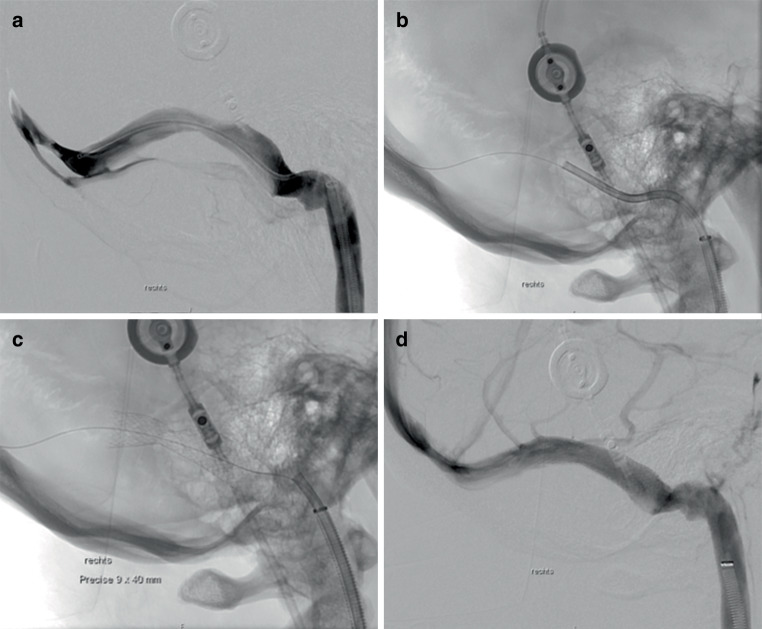
**a** Shows the digital subtraction angiography (DSA) of a patient who has idiopathic intracranial hypertension with an extrinsic stenosis of the right transverse sinus that has generated a transstenotic pressure gradient of 24 mm Hg measured by venous sinus manometry. Advancing the Precise Pro RX carotid stent (9 × 40 mm) turned out to be difficult owing to the kinked anatomy of the sigmoid sinus and the stiffness of the stent system (**b**). Successful stent delivery was only achieved after advancing an 8F Guider Softip over a Vasco +35 intermediate catheter through an 8F arrow sheath into the transverse sinus. **c** Shows residual stenosis with resolution after percutaneous transluminal angioplasty (**d**)

Stent delivery was easier and more successful with the CP than with the PP (difficult or failed stent delivery 0.0% versus 57.1%). The number of attempts was lower in the CP than in the PP group (≥ 2: 0.0% versus 40.0%). Additional balloon dilatation due to residual stenosis was required in 3/10 CP patients and in 2/5 PP patients (30% versus 40%). No periinterventional complications were noted during VSS. None of the patients showed stent thrombosis, venous occlusion or stenosis requiring retreatment at 6‑month follow-up. Interventional characteristics are listed in Table 4. Figures 1 and 2 show the successful deployment of the CP and the PP in IIH patients with venous sinus stenosis.

**Table 4:** Difficulty of stent delivery and periinterventional and postinterventional outcome parameters. Data are expressed as percentages (*n*)

		All patients	Casper^TM^ RX stent group	Precise Pro RX^TM^ stent group
**Difficulty of stent delivery (per attempt)**	**100% (17/17)**	–	–	–
Easy	–	76.5% (13/17)	100% (10/10)	42.9% (3/7)
Difficult or failed	–	23.5% (4/17)	0.0% (0/10)	57.1% (4/7)
**Attempts (per patient)**	**100% (15/15)**	–	–	–
<2	–	86.7% (13/15)	100% (10/10)	60.0% (3/5)
≥2	–	13.3% (2/15)	0.0% (0/10)	40% (2/5)
**Other outcome parameters**	**–**	–	–	–
Additional balloon dilatation applied due to residual stenosis	100% (15/15)	33.3% (5/15)	30.0% (3)	40.0% (2)
Symptomatic intracranial hemorrhage	100% (15/15)	0.0% (0/15)	0.0% (0/10)	0.0% (0/5)
Venous infarction	100% (15/15)	0.0% (0/15)	0.0% (0/10)	0.0% (0/5)
Other complications	100% (15/15)	6.7% (1/15)	0.0% (0/10)	20.0% (1/5)

### Headache Before and After VSS

Four of 10 patients later treated with the CP and 3 of 5 patients who received the PP complained of multiple types of headaches before the intervention [20] (40.0% versus 60.0%; *p* = 0.408). Headache intensity was initially significantly higher among patients treated with the CP (VAS 8.75 versus 6.25, *p* = 0.048), whereas headache frequency before VSS was similar in the two groups (CP versus PP; 7 times per week versus 7 times per week, *p* = 0.933). Patients in both groups showed a substantial but similar reduction of headache intensity (CP versus PP; headache VAS after VSS: 0 versus 0, *p* = 0.808; headache VAS improvement after VSS: −8 versus −5.5, *p* = 0.087). Headache frequency also decreased in both groups (CP versus PP; headache frequency after VSS: 0 versus 0, *p* = 0.808; headache frequency improvement after VSS: −7 times per week versus −7 times per week, *p* = 0.920) after VSS.

### Other IIH-related Symptoms Before and After VSS

All patients included in this study had additional IIH-related symptoms of which visual disturbances (*n* = 11/15; 73.3%), nausea/vomiting (*n* = 10/15; 66.7%) and tinnitus (*n* = 10/15; 66.7%) were the most frequent. Papilledema was documented in 6/10 patients treated with the CP and 4/5 treated with PP (60.0% versus 80.0%; *p* = 0.439). All patients who were available for interview 6 months after VSS (*n* = 12/15; 80.0%) reported substantial impairment of daily life before VSS, which they attributed to the restraints imposed by IIH. Symptom duration prior to any treatment tended to be longer but was not significantly different between patients treated with the CP and the PP (26.9 months versus 12 months; *p* = 0.953). All patients who reported nausea/vomiting (*n* = 10/10), photophobia/phonophobia (*n* = 4/4) or diplopia (*n* = 3/3) showed substantial improvement or complete cessation of these complaints after VSS. Almost all patients who had tinnitus (*n* = 7/9; CP versus PP: 100% versus 50.0%, *p* = 0.073) or visual disturbances other than diplopia (*n* = 10/11; CP versus PP: 100% versus 80.0%, *p* = 0.250) reported substantial improvement or complete cessation of those symptoms. All patients with papilledema who were available for follow-up (*n* = 8/9) showed substantial or complete resolution on ophthalmological examination. A substantial improvement in quality of life after VSS was reported by all patients who were available for interview (*n* = 12/15).

### Pressure Values Before and After VSS

No significant differences in CSF OP for patients treated with CP versus PP before (35 cmH_2_0 versus 27 cmH_2_0; *p* = 0.414) and after VSS (22.0 cmH_2_0 versus 20 cmH_2_0, *p* = 0.373) were found. Similarly, TSPG under LA and GA before (TSPG under LA: 23 mm Hg versus 21.5 mm Hg, *p* = 0.733; TSPG under GA: 8.5 mm Hg versus 19 mm Hg, *p* = 0.099) and after VSS (TSPG in LA: 1.5 mm Hg versus 0 mm Hg, *p* = 0.454; TSPG in GA: 1 mm Hg versus 1 mm Hg, *p* = 0.953) were not significantly different between the CP and PP groups. Both stents achieved notable and comparable reduction of CSF OP (−12 cmH_2_0 versus−10 cmH_2_0, *p* = 1.000), TSPG under LA (−19 mm Hg versus −21 mm Hg, *p* = 0.304) and TSPG under GA (−8 mm Hg versus −19 mm Hg, *p* = 0.165).

### Clinical Outcome Parameters

No patients in either group had any new neurological deficits, symptomatic intracranial hemorrhage or venous infarction after VSS. One patient who received the PP developed an abscess at the puncture site of the femoral arterial access, which led to sepsis. The patient was treated with antibiotics and recovered well. The interval between VSS and last clinical follow-up tended to be longer in the PP group, but this difference was not significant (17.1 months versus 31.7 months, *p* = 0.126).

## Discussion

The main findings of this study were: (1) stent delivery was easier and more successful with the CP than with the PP. (2) No severe periinterventional or postinterventional complications occurred in either group. (3) None of the patients showed stent thrombosis, venous occlusion or stenosis requiring retreatment at 6‑month follow-up. (4) The CSF OP, TSPG improvement immediately after VSS and TSPG 6 months after stenting were similar in the CP and the PP group. (5) Both groups showed substantial and comparable reduction of headache intensity and frequency after VSS. (6) Almost all patients who had other IIH-related symptoms showed either improvement or complete cessation of the complaints after VSS, resulting in a comparable and substantial improvement in daily life.

To the best of our knowledge this is the first study to examine the benefits of using the CP for VSS in IIH patients who have venous sinus stenosis. It is also the first attempt to compare different stent types used for VSS with respect to difficulty of delivery, technical and clinical outcome as well as complication rates [17]. Our data support the hypothesis that stent-specific characteristics influence the technical success of stent delivery.

Navigating the intracranial venous system and crossing the venous stenosis for safe stent delivery may be challenging for several reasons. These include the tortuosity of the transverse and sigmoid sinuses, the high degree of stenosis, the angle of the stenotic segment, small venous channels within the sinus, arachnoid granulations, fibrous trabeculae or the presence of a cortical vein draining into the dural sinus, which are frequently observed in IIH patients [10–13]. Inability to advance the stent system beyond the venous stenosis can result in failure of the procedure.

Several endovascular techniques that may help to manage these challenging circumstances have been reported. For example, using stiffer microwires and microcatheters combined with an increased forward pressure to the catheter system may lead to technical success; however, forceful device maneuvers, especially in patients on antiplatelet therapy, can lead to severe hemorrhagic complications due to avulsion of cortical veins, dissection or perforation of the sinus [14–16]. The rising number of IIH patients being treated with VSS means that the probability of encountering these relatively rare complications may increase too. Intracranial hemorrhages resulting from venous or cortical vein injury are particularly difficult to treat and may cause severe morbidity or even death. Choosing the best techniques and applying appropriate materials may decrease the risk of these complications. Schwarz et al. [10] described the cobra technique as favorable when navigation and/or advancement of an intermediate catheter beyond the stenosis requires increased loading of the system: A 3.5 mm balloon is advanced through the intermediate catheter to the level of the stenosis and inflated below the balloon’s nominal pressure. The intermediate catheter is then advanced in close proximity to the proximal end of the balloon placing it partially within the intermediate catheter. Finally, the balloon and intermediate catheter can be advanced together over the microwire and beyond the stenotic segment. Gordon et al. [11] described transverse-sigmoid sinus stenting from the contralateral dural sinus as an alternative to the typical antegrade approach. This seems to be advantageous in the case of a high-grade stenosis or whenever the angle at which the tip of the stent delivery catheter entered the transverse sinus prevents advancement of the stent system in an antegrade way. Delivering the stent from the contralateral side changes the angle of entry and may allow advancement of the stent without injuring the vessel wall; however, this approach is only possible in patients with a contralateral sinus that is large enough to accommodate the stent delivery system and a superior sagittal sinus bifurcation that is not too high. Thus, stent delivery systems that could provide better navigability and flexibility may improve the chances of technically successful VSS.

The unique flexibility of the CP makes it particularly suitable for patients with tortuous intracranial venous anatomy; however, the inverse relationship between device flexibility and radial force is a limiting factor that needs to be considered to guarantee long-term stent patency [25]. Despite concerns about increased thrombogenicity caused by the CP’s larger contact surface, no venous thrombosis, occlusion or infarction was observed in any of the patients. Its low-profile delivery system provides better crossability, making lesions and tortuous vessel segments more accessible for interventional maneuvering.

Leishangthem et al. [17] reported that the stents most frequently used for VSS are the PP and the Carotid Wallstent^TM^ (Boston Scientific, Marlborough, MA, USA). Although the challenges of safe stent delivery are well-known [10], many studies do not mention the rationale for stent selection nor do they identify the particular stents chosen [17, 22]. Further research is required to define stent characteristics beneficial for the treatment of venous sinus stenosis in patients with IIH. The development of IIH-specific stents could improve technical efficacy of VSS. Ultimately this would help to avoid aggressive maneuvers that could lead to severe complications [14–16] and increase the number of patients showing sustainable long-term improvement after VSS.

Our data also underline the safety and efficacy of VSS in IIH patients with venous sinus stenosis [4–9]. Other than VSS, few treatment options are available for patients who do not respond adequately to conservative treatment [18]. The VSS has proven to be a safe and efficient alternative that does not seem to be inferior to surgical treatment methods [3, 26]. Given the young age of patients with IIH, VSS constitutes an appealing treatment option that promises a good chance of symptom improvement and an excellent outcome.

The reduction of CSF OP and TSPG after VSS, followed by a substantial improvement of IIH-related symptoms, suggest that changes in intracranial venous pressure play a central role in the pathomechanisms causing IIH-related symptoms and may help categorize IIH patients based on objective phenotypes [26–28]. Although IIH genesis is not yet fully understood, the regulation of intracranial venous pressure achieved by VSS seems to offer an effective approach for treatment of the wide spectrum of IIH-related symptoms.

### Limitations

This was a retrospective, monocentric study, which may limit generalizability. Only 15 patients were included, which could have led to sampling error. All data presented in this study were gathered either by extensive chart review and/or patient interviews; however, some patients were referred by external physicians who could not always provide the information required for our analysis. Documentation of clinical information before and after stenting was not standardized and was performed by different departments. In some instances, patient interviews were conducted up to 3.75 years after VSS. Thus, results on symptom improvement are prone to recall bias as some patients may overestimate or underestimate symptoms prior to VSS. As symptom improvement is limited by initial severity and almost all patients reported substantial improvement or complete resolution of IIH-related symptoms, correlation analyses between symptom improvement and duration of the follow-up period were not informative. Some patients were lost to follow-up causing further data gaps and potential selection bias. Like any symptoms, IIH-related complaints and daily life impairment are subjective and hard to quantify. Hence, headache was the only symptom to be quantified using the VAS.

## Conclusion

We observed that the CASPER^TM^ carotid stent is a safe and effective alternative for the treatment of venous sinus stenosis in IIH patients that do not respond to conservative treatment. It may reduce the risk of difficult or failed stent delivery in patients with challenging intracranial venous anatomy. Our findings underline the need for dedicated stent systems that are adapted to the tortuous intracranial venous anatomy frequently observed in IIH patients requiring treatment for venous sinus stenosis.

## Supplementary Information

The patient questionnaire regarding the improvement of IIH-related symptoms after venous sinus stenting is provided as Supplement Form A.

## Abbreviations

CP: Casper^TM^ RX stent
CSF OP: Cerebrospinal fluid opening pressure
DSA: Digital subtraction angiography
GA: General anesthesia
IIH: Idiopathic intracranial hypertension
LA: Local anesthesia
PP: Precise Pro RX^TM^ stent
TSPG: Transstenotic pressure gradient
VSM: Venous sinus manometry
VSS: Venous sinus stenting
