# Correction to: Casper Versus Precise Stent for the Treatment of Patients with Idiopathic Intracranial Hypertension

**DOI:** 10.1007/s00062-021-01055-9

**Published:** 2021-07-13

**Authors:** Nebiyat F. Belachew, Severin Baschung, William Almiri, Ruben Encinas, Johannes Kaesmacher, Tomas Dobrocky, Christoph J. Schankin, Mathias Abegg, Eike I. Piechowiak, Andreas Raabe, Jan Gralla, Pasquale Mordasini

**Affiliations:** 1 Department of Diagnostic and Interventional Neuroradiology, Inselspital, Bern University Hospital, and University of Bern, Freiburgstraße 18, 3010 Bern, Switzerland; 2 Faculty of Medicine, University of Bern, Bern, Switzerland; 3 Department of Diagnostic, Interventional and Pediatric Radiology, Inselspital, Bern University Hospital, and University of Bern, Bern, Switzerland; 4 Department of Neurology, Inselspital, Bern University Hospital, and University of Bern, Bern, Switzerland; 5 Department of Ophthalmology, Inselspital, Bern University Hospital, and University of Bern, Bern, Switzerland; 6 Department of Neurosurgery, Inselspital, Bern University Hospital, and University of Bern, Bern, Switzerland

**Correction to:**

**Clin Neuroradiol 2021**

10.1007/s00062-021-01024-2

After the article was published online, the abstract was revised again. The correct abstract is given below. The original article has been corrected.

## Abstract

### Purpose

We hypothesized that due to its specific characteristics, the Casper^TM^ RX carotid stent (CP) might be particularly suitable for venous sinus stenting (VSS) in patients with idiopathic intracranial hypertension (IIH). To test this theory, we compared it to the commonly used Precise Pro RX^TM^ stent (PP).

### Methods

A total of 15 patients with IIH (median age 28.7 years) were reviewed retrospectively. Technical aspects as well as peri- and postinterventional complication rates were examined in patients treated with CP (*n* = 10) and the PP (*n* = 5). Improvements in cerebrospinal fluid opening pressure (CSF OP), transstenotic pressure gradient (TSPG) and clinical symptoms were also assessed.

### Results

Stent delivery was easier and more successful with the CP than the PP (difficult/failed stent delivery 0.0% versus 57.1%) and consequently achieved with less attempts (≥ 2: 0.0% versus 40.0%). No severe peri- or postinterventional complications or instances of in-stent thrombosis and/or stenosis were observed during follow-up. Improvement of CSF OP and TSPG immediately after VSS as well as at 6‑month follow-up was comparable between the CP and PP group. Both groups showed substantial and similar decreases in intensity and frequency of headache. Almost all patients with other IIH-related symptoms showed either improvement or complete resolution of those symptoms after VSS. All patients who were available for interview (*n* = 12/15) reported a substantial improvement in quality of life.

### Conclusion

VSS using the CP seems to be safe and effective. The CP may reduce the risk of difficult or failed stent delivery in patients with challenging intracranial venous anatomy.

