# Supplementary material for: Casper Versus Precise Stent for the Treatment of Patients with Idiopathic Intracranial Hypertension
Source: Clin Neuroradiol. 2021 May 18;31(3):853–62. doi: 10.1007/s00062-021-01024-2 (PMC8463398; doi:10.1007/s00062-021-01024-2)
Supplement: Supplementary file 1 — The patient questionnaire regarding the improvement of IIH-related symptoms after venous sinus stenting is provided as Supplement Form A. [file 62_2021_1024_MOESM1_ESM.pdf]

Supplement: Casper Carotid Stent for the Treatment of Venous Sinus Stenosis in Patients with Idiopathic Intracranial Hypertension

Nebiyat F. Belachew, MD[1]; Severin Baschung[2]; William Almiri, MD[1]; Ruben Encinas, MD[1]; Johannes Kaesmacher, MD[1,3]; Tomas Dobrocky, MD[1]; Christoph J. Schankin, MD[4]; Mathias Abegg, MD[5]; Eike I. Piechowiak, MD[1]; Andreas Raabe, MD[6]; Jan Gralla, MD, MSc[1]; Pasquale Mordasini, MD, MSc[1]

[1] Department of Diagnostic and Interventional Neuroradiology, Inselspital, Bern University Hospital, and University of Bern, Switzerland

[2] Faculty of Medicine, University of Bern, Switzerland

[3] Department of Diagnostic, Interventional and Pediatric Radiology, Inselspital, Bern University Hospital, and University of Bern, Switzerland

[4] Department of Neurology, Inselspital, Bern University Hospital, and University of Bern, Switzerland

[5] Department of Ophthalmology, Inselspital, Bern University Hospital, and University of Bern, Switzerland

[6] Department of Neurosurgery, Inselspital, Bern University Hospital, and University of Bern, Switzerland

Corresponding author:

Nebiyat Filate Belachew, MD

ORCID iD: https://orcid.org/0000-0001-5285-5922

Department of Diagnostic and Interventional Neuroradiology

Inselspital

Freiburgstrasse 18

CH-3010, Switzerland

E-Mail: nebiyatfilate.belachew@insel.ch

Phone: +41 31 632 26 55

Supplementary Form A

Patient Questionnaire Regarding the Improvement of IIH-related Symptoms after Venous Sinus Stenting

A: Do you consent to this interview and the use of any information you give for research purposes?

1. Did you experience headaches prior to venous sinus stenting? (If answer is “yes”, continue with questions 1A–1E; if answer is “no” continue with question 2).
   - 1A. Before the intervention, would you say that you experienced more than one type of headache (in terms of development, localization, expansion, severity, time of occurrence etc.)?
   - 1B. Did one of these types of headache change (in terms of intensity and frequency of occurrence) after venous sinus stenting? If you had more than one type of headache before venous stenting, please refer to the type that changed after venous sinus stenting when you answer questions 1B–1E. If no change was noticed, refer to the type that is most likely caused by increased intracranial pressure (i.e. severe, migraine-like/throbbing headache accompanied by visual disturbances or tinnitus).
   - 1B. On a scale from 0 to 10, where would you rank the intensity of headaches before the intervention?
   - 1C. How many times per week did you experience headaches before the intervention?
   - 1D. On a scale from 0 to 10, where would you rank the intensity of the headache after the intervention?
   - 1E. How many times per week did you experience headaches after the intervention?
2. Did you experience any nausea or vomiting before the intervention? Was there any substantial improvement after venous sinus stenting?
3. Did you experience any photophobia or phonophobia before the intervention? Was there any substantial improvement after venous sinus stenting?
4. Did you experience any tinnitus before the intervention? Was there any substantial improvement after venous sinus stenting?
5. Did you experience any diplopia before the intervention? Was there any substantial improvement after venous sinus stenting?
6. Did you experience any visual disturbances before the intervention? Was there any substantial improvement after venous sinus stenting?
7. How would you describe the impairment of your daily life before the intervention: “severe”, “mild” or “non-existent”?
8. How would you describe the impairment of your daily life after the intervention: “severe”, “mild” or “non-existent”?
9. In your opinion, has your quality of life/daily activity improved substantially as a consequence of undergoing venous sinus stenting?

Date, Location:

Note: Information gathered from this questionnaire was used to supplement the data drawn from the clinical information system of Bern University Hospital and medical records provided by treating physicians.
